# Azimuthal-rotation sample holder for molecular orientation analysis

**DOI:** 10.1107/S160057752000990X

**Published:** 2020-08-18

**Authors:** Takayuki Harano, Yasuo Takeichi, Takuji Ohigashi, Daisuke Shindo, Eiji Nemoto, Daisuke Wakabayashi, Shohei Yamashita, Reiko Murao, Masao Kimura

**Affiliations:** a Advanced Technology Research Laboratories, Research and Development, Nippon Steel Corporation, 20-1 Shintomi, Futtsu, Chiba 293-8511, Japan; b Department of Materials Structure Science, School of High Energy Accelerator Science, The Graduate University for Advanced Studies (SOKENDAI), 1-1 Oho, Tsukuba, Ibaraki 305-0801, Japan; c Photon Factory, Institute of Materials Structure Science, High Energy Accelerator Research Organization (KEK), 1-1 Oho, Tsukuba, Ibaraki 305-0801, Japan; d UVSOR Synchrotron Facility, Institute for Molecular Science, 38 Nishigonaka, Myodaiji, Okazaki, Aichi 444-8585, Japan; e Department of Functional Molecular Science, School of Physical Sciences, The Graduate University for Advanced Studies (SOKENDAI), 38 Nishigonaka, Myodaiji, Okazaki, Aichi 444-8585, Japan; f Kohzu Precision Co. Ltd, 2-6-15 Kurigi, Asao, Kawasaki, Kanagawa 215-8521, Japan

**Keywords:** scanning transmission X-ray microscopy, X-ray absorption near edge, orbital orientation, chemical structure, carbon

## Abstract

In this study, an azimuthal-rotation sample holder compatible with scanning transmission X-ray microscopy was developed. This holder exhibits improvement in the accuracy of rotation angles and reduces the displacement of the rotation axes during azimuthal rotation by using a crossed roller bearing. To evaluate the performance of the holder, the authors investigated the dependence of the optical density around the C *K*-edge absorption of π-orbital-oriented domains in natural spherical graphite on the rotational angle by using linearly horizontally and vertically polarized undulator radiation. Based on the dependence of the optical density ratio between C 1*s* → π* and 1*s* → σ* excitation on the polarization angle of the X-rays, the average two-dimensional orientation angle of the π orbital in each position in a natural spherical graphite sample was visualized.

## Abstract 2

An azimuthal-rotation sample holder, with improvements in the rotation-angle accuracy and rotation-axes displacement during azimuthal rotation, was developed for molecular-orientation analysis using scanning transmission X-ray microscopy.

## 1. Introduction   

The physical properties of carbon materials, such as rubber, polymer-blend resins, carbon fibers, and carbon fiber-reinforced plastics, are based not only on the domain shape and size of each phase but also on the spatial distribution and orientation of their chemical structures (functional groups and chemical bonding) in each phase (Miller *et al.*, 1987 ▸; Otani & Oya, 1986 ▸; Soo-Jin, 2018 ▸). Scanning transmission X-ray microscopy (STXM) is used to observe the distribution of chemical structures with a spatial resolution higher than ∼50 nm (Hitchcock, 2012 ▸). The X-ray absorption (XA) intensity [optical density (OD) is generally used for STXM measurements] reflects the interaction between the polarization of X-rays and the orientation of molecular or chemical bonding orbitals (Hernández-Cruz *et al.*, 2007 ▸; Harano *et al.*, 2017 ▸; Watts *et al.*, 2011 ▸; Watts & Ade, 2008 ▸). Hence, through STXM, the distribution of orbital orientation can be determined by observing the variations in the OD contrast within the field-of-view (FOV) when the sample is rotated at multiple angles in the azimuthal direction of the incident X-rays (Ohigashi *et al.*, 2016 ▸; Watts *et al.*, 2011 ▸; Watts & Ade, 2008 ▸). With the previous holders, the focus depth of the Fresnel zone plate (FZP) and the FOV of the observations were around the micrometre order, and maintaining the error motion within that range was difficult. In this study, an azimuthal-rotation sample holder was developed, which exhibited improvements in rotation accuracy and reduction in the displacement of the rotation axis during azimuthal rotation. Thus, the dependence of OD on the rotational angle was measured to investigate the domain derived from π-orbitals (carbon double bonds) in natural spherical graphite. Furthermore, the performance of the developed azimuthal-rotation sample holder was evaluated.

## 2. Experimental   

**Figure 1:**
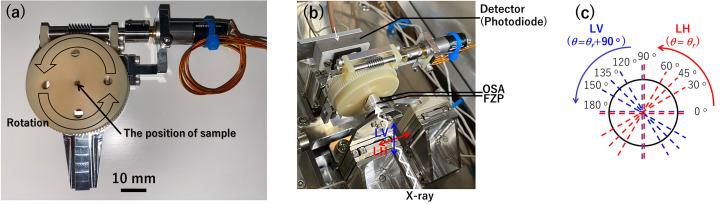
(*a*) Azimuthal-rotation sample holder developed for cSTXM. (*b*) Photograph of the holder used in cSTXM. (*c*) Direction of the electric field in the LH and LV modes applied to the sample during the measurements (viewed from the detector side).

Fig. 1 ▸(*a*) shows the developed azimuthal-rotation sample holder, with a weight of 90 g and thickness of 19.6 mm. The part of the holder attached to the STXM chamber and the part supporting the motor were fabricated using aluminium alloy (A6061) and aromatic polyamide resin, respectively. The worm shaft was made of SUS416. The sample mounted on the center of the disk plate was fabricated using aromatic polyamide resin and can be rotated 360° with a minimum rotation angle of 0.002° by using a small two-phase stepping motor (AM1020RC018008+10/1; Faulhaber Co. Ltd), which is vacuum compatible. The aforementioned holders also use the same motor. The pulse signal sent from the controller (Tsuji Electronics Co. Ltd) is converted into the drive signal by the motor driver (Faulhaber Co. Ltd), as explained in previous works (Hernández-Cruz *et al.*, 2007; Ohigashi *et al.*, 2016 ▸). It is critical to ensure that the position of the sample is maintained in such a manner that it stays in focus during the sample rotation. Thus, the rotation was supported by a crossed roller bearing (REV2008CC0P5S, THK Co. Ltd), which reduces backlash or errors when rotating the sample holder. The developed system could solve the problems that often occur owing to the brass bevel gear and conventional ball bearing, which has been used in previous works (Hernández-Cruz *et al.*, 2007 ▸; Ohigashi *et al.*, 2016 ▸), *i.e.* the slip in rotating axis during sample rotation, particularly when the sample position is displaced. The radial and axial runout tolerances were less than 5 µm/360° and less than 7 µm, respectively. Thus, the developed holder can be used to rotate the specimens with a high angle accuracy.

Furthermore, STXM measurements were conducted using BL-19A at the Photon Factory [Institute of Materials Structure Science, High Energy Accelerator Research Organization (KEK), Japan]. Fig. 1 ▸(*a*) shows a photograph of the developed azimuthal-rotation sample holder for the compact STXM (cSTXM) (Takeichi *et al.*, 2016 ▸). Fig. 1 ▸(*b*) shows a photograph of the holder loaded in the cSTXM. The measurement mode corresponded to the transmission mode. The spatial resolution of this technique improves by more than 50 nm with the application of a FZP. The order-sorting aperture (OSA) is located between the FZP and sample. In this study, a photodiode was used as the detector. In addition, the APPLE-II type undulator of BL-19 can generate synchrotron radiation X-rays with linear horizontal (LH) and liner vertical (LV) polarization modes (Sasaki *et al.*, 1993 ▸). To investigate the molecular orientation, measurements must be performed using X-ray absorption near-edge spectroscopy (XANES) with not just two polarizations of the X-ray but also other polarization directions via sample rotation. Hence, by switching the polarizations (LH or LV) and rotating the sample (θ_r_ = 0°–90°), the polarization angles (θ) of the electric field of the synchrotron radiation X-rays with respect to the sample can be changed from 0° to 180° [Fig. 1 ▸(*c*)] as follows: θ = θ_r_ + 0° (LH) or θ_r_ + 90° (LV). These polarizations enable the investigation of two types of relationships between the sample and electric field of the X-ray at the same rotation angle. This can reduce the total measurement by half or more. The *aXis2000* software was used to analyze the image stack data (a set of XA images of a sequence of synchrotron radiation X-ray energies) (Hitchcock, 2009 ▸). Fig. 1 ▸(*c*) shows the direction of the electric field in the LH and LV modes when viewed from downstream.

A natural spherical graphite sample was used for the STXM measurements. The sample was sliced into thin films through focused ion-beam (FIB) processing. Before slicing, the sample surface was coated with Pt to avoid damage due to the ion beam. The thickness of the sample was adjusted to approximately 50 nm through argon milling.

## 3. Results and discussion   

**Figure 2:**
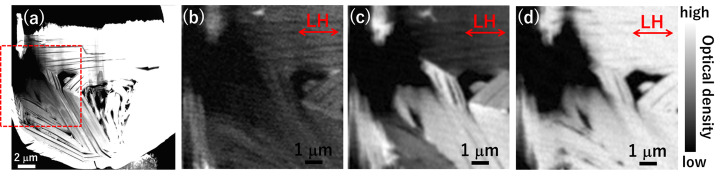
(*a*) TEM (dark field) image of natural spherical graphite. The red dotted line shows the FOV of XA images obtained via cSTXM with a rotation angle of θ = 0°. XA images at θ = 0° (LH) with energies of (*b*) 280.0, (*c*) 285.7, and (*d*) 292.0 eV. The red arrow indicates the X-ray polarization directions of LH.

Fig. 2 ▸ shows a transmission electron microscopy (TEM) image and XA images of the natural spherical graphite sample. In the TEM image [Fig. 2 ▸(*a*)], the red dotted line shows the FOV of the XA image obtained by setting the cSTXM at θ = 0° (θ_r_ = 0°, LH). Figs. 2 ▸(*b*), 2(*c*), and 2(*d*) present XA images at θ = 0° (θ_r_ = 0°, LH) with different energies corresponding to 280.0 eV (pre-edge), 285.7 eV (1*s* → π*), and 292.0 eV (1*s* → σ*), respectively. The contrast in the XA images represents OD, which is dependent on the difference in energy among locations. Thus, the chemical structure and orientation of the π-orbitals varies with respect to location. The OD exhibits its maximum value when the direction of the π-orbital is parallel to that of the polarization (Brandes *et al.*, 2008 ▸).

**Figure 3:**
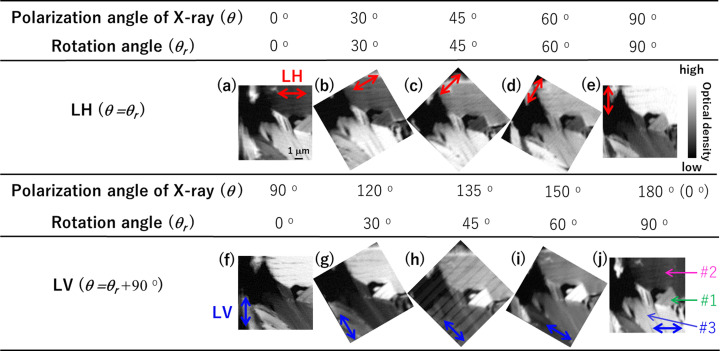
XA images (OD contrast) of natural spheroidal graphite at 285.7 eV, measured at a wide range of polarization angles, θ = 0°–180° for the LH and LV modes; the FOV is 9 µm × 9 µm with a pixel size of 90 nm. The red and blue arrows indicate the X-ray polarization directions of LH and LV, respectively.

Fig. 3 ▸ shows XA images of the natural spheroidal graphite at 285.7 eV, measured at a wide range of angles from θ = 0° to θ = 180° with two linearly polarized X-rays (LH and LV). Furthermore, the FOV was 9 µm × 9 µm with a pixel size of 90 nm. The dwell time of X-ray detection at each pixel was 40 ms.

**Figure 4:**
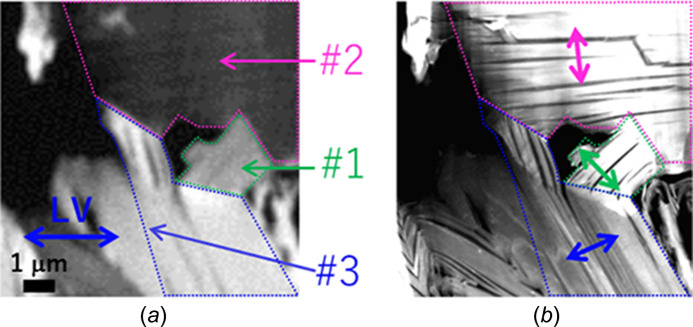
(*a*) XA images (OD contrast) of natural spheroidal graphite at 285.7 eV [Fig. 3 ▸(*j*)]. (*b*) TEM image of the FOV in (*a*). Dotted areas (#1, #2, and #3) show π-orbital-oriented domains determined via cSTXM. The average orientation angles of domains #1, #2, and #3 are 134.3°, 96.5°, and 24.2°, respectively.

**Figure 5:**
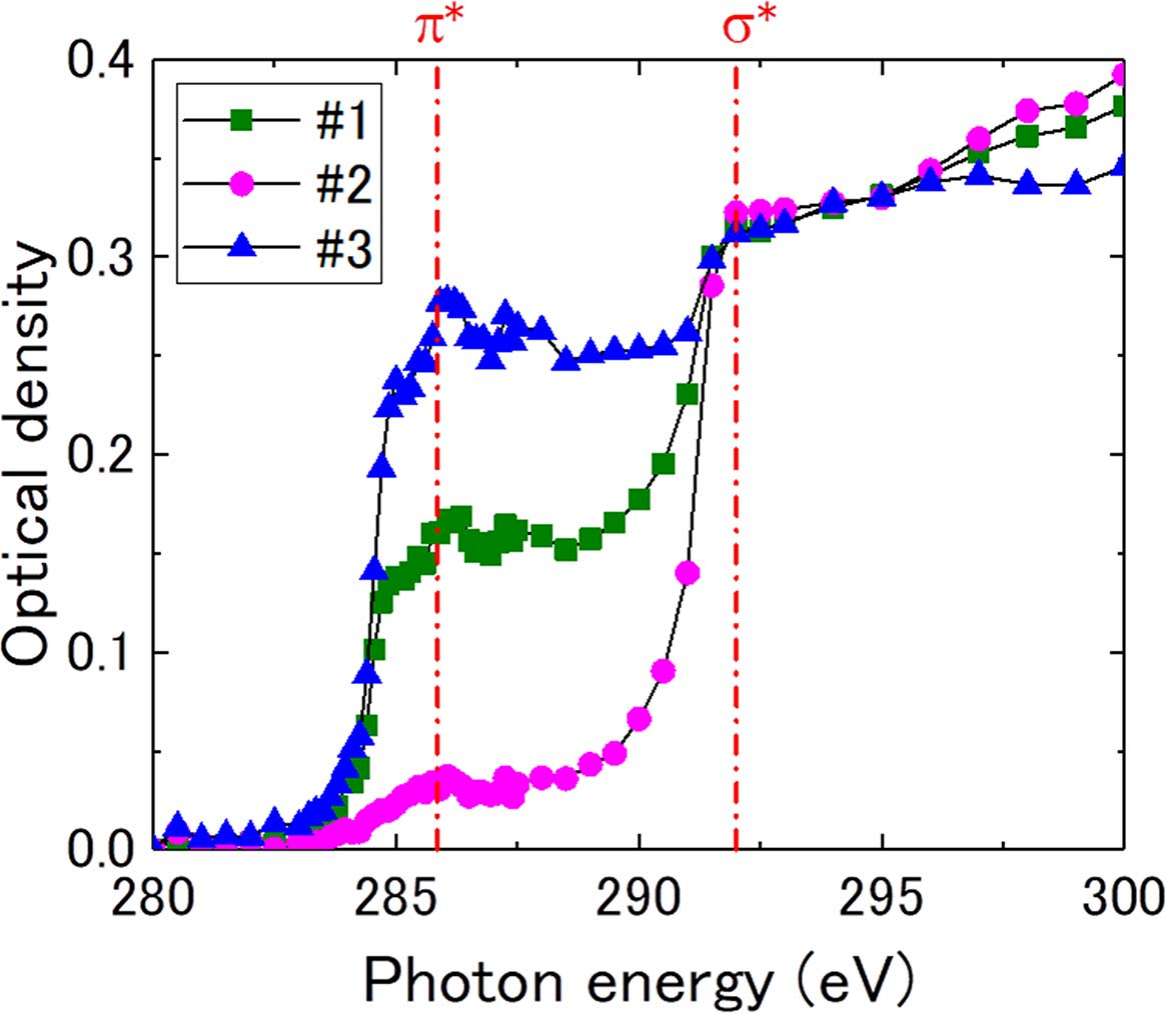
C *K*-edge spectra in domains #1, #2, and #3 for condition (*j*) in Fig. 3 ▸.

**Figure 6:**
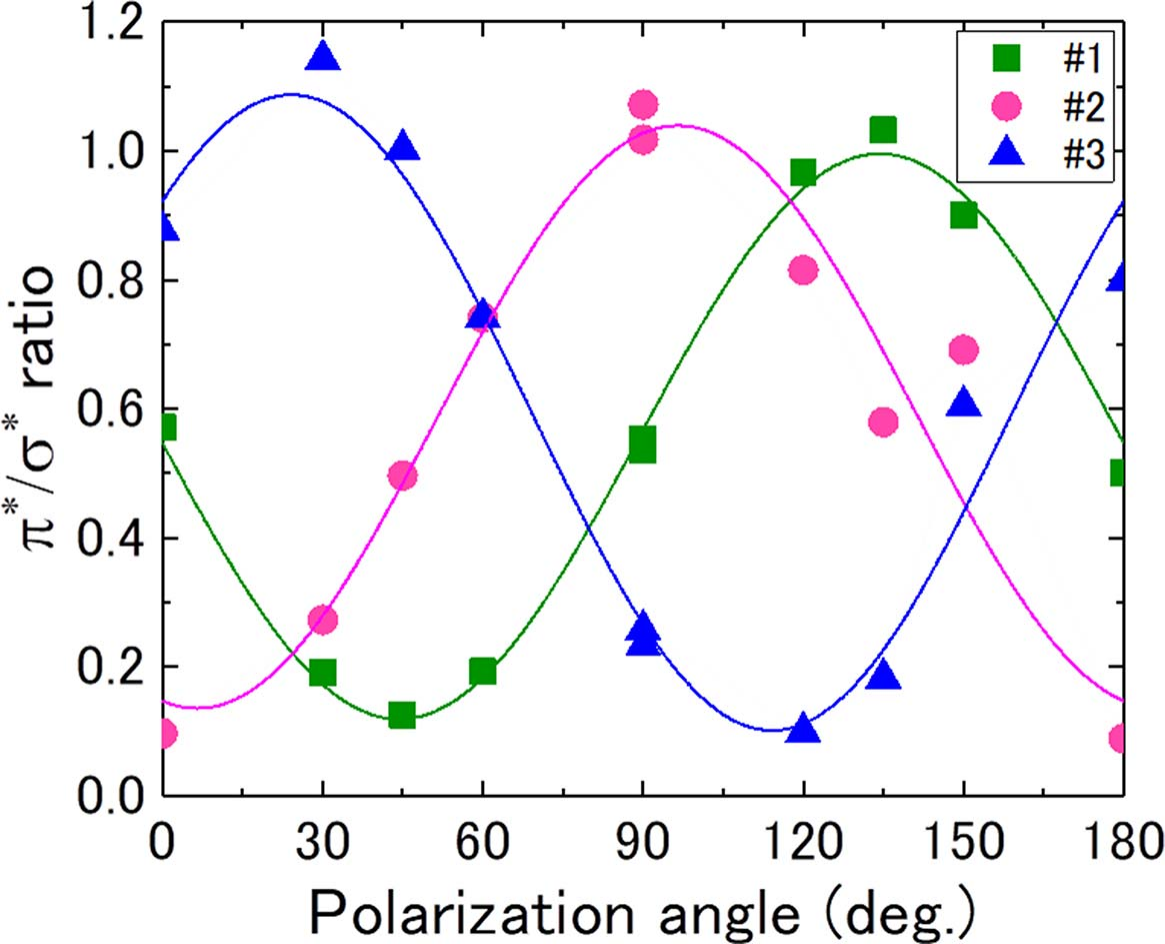
Rotational dependence of the π^*^/σ^*^ ratio (OD ratios at 285.7 and 292.0 eV) in areas #1, #2, and #3 shown in Fig. 3 ▸(*j*). The three solid-line curves are the results of the nonlinear fitting based on equation (1).

Fig. 4(*a*) ▸ shows XA images of natural spheroidal graphite at 285.7 eV, which is similar to that in Fig. 3 ▸(*j*), and Fig. 4 ▸(*b*) shows a TEM image of the FOV in Fig. 4 ▸(*a*). The dotted areas (#1, #2, and #3) of the π-orbital orientation were determined via cSTXM through the following procedure. Cleavage cracking in natural spheroidal graphite, as shown in Fig. 4 ▸(*b*), was observed in each area. These cracks face a certain direction in each OD region, indicating traces of separation between the graphene sheets and suggesting that these domains exhibit different orientations in the π-orbital domains. A quantitative relationship of the orientation was investigated using STXM data. Fig. 5 ▸ shows the C *K*-edge XANES spectra in the three areas (#1, #2, and #3) shown in Fig. 4 ▸(*a*) at θ = 180°. The OD intensity at *E* = 285.7 eV changes significantly among the domains, indicating the difference in the relationship between the polarization angle and direction of the π orbital. It is noted that these spectra are weaker and broader than those of highly oriented pyrolytic graphite (HOPG) (Watts & Ade, 2008 ▸). This is because natural spherical graphite might be more sensitive to FIB and argon milling and the crystallinity of graphite in natural spherical graphite is lower than that of HOPG. In addition, these spectra might be affected by the sample oxidization. Fig. 6 ▸ shows the rotational dependence of the π^*^/σ^*^ ratio {the ratio between OD at 285.7 eV [*I*(1*s* → π*)] and at 292.0 eV [*I*(1*s* → σ*)]} on the polarization angle, θ, in areas #1, #2, and #3 shown in Fig. 4 ▸(*a*). Hence, the OD contrast clearly changes with respect to the polarization angle, θ. Furthermore, the contrasts of (*a*) and (*j*) as well as (*e*) and (*f*) in Fig. 3 ▸ show the same relationship in terms of the sample position and direction of the electric field of the X-ray. Thus, the change in OD intensity at *E* = 285.7 eV (1*s* → π*) is proportional to cos^2^θ, as theoretically stated by Stöhr (1992 ▸). In this case, π^*^/σ^*^ is the ratio between *I*(1*s* → π*) and *I*(1*s* → σ*) and is defined as follows,
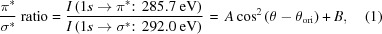
where *A*, θ_ori_, and *B* denote the amplitude of OD vibration, orientation angle of the π orbital, and background constant, respectively. A dichroic response of 30% at 292.0 eV was reported for HOPG (Watts & Ade, 2008 ▸). However, in the case of natural spherical graphite in this study, we found that the optical densities at 292.0 eV in three different areas (#1– #3) were identical as shown in Fig. 5 ▸. The dichroic response at this energy, if it exists, can slightly deform the sinusoidal rotational response but has little effect on the spatial distribution of *A*, θ_ori_, and *B* in equation (1). Therefore, we judged that the OD intensity at 292.0 eV can be used to normalize the 1*s* → π* intensity.

Based on the nonlinear fitting of equation (1), the three solid-line curves are shown in Fig. 6 ▸. Thus, the average orientation angles of domains #1, #2, and #3 in Fig. 4 ▸(*a*) were obtained as 134.3°, 96.5°, and 24.2°, respectively [Fig. 4 ▸(*b*)]. The standard deviation/corrected *R* ^2^ of the average orientation angles of domains #1, #2, and #3 were 0.95°/0.99, 3.1°/0.90, and 2.3°/0.95, respectively. By using the aforementioned procedure, the π^*^/σ^*^ ratio can be calculated at each position of the sample.

**Figure 7:**
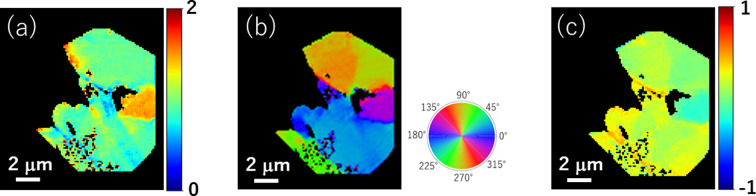
Maps of (*a*) amplitude (*A*), (*b*) orientation angle (θ_ori_), and (*c*) background (*B*) in natural spherical graphite.

Figs. 7 ▸(*a*), 7(*b*), and 7(*c*) show maps of *A*, θ_ori_, and *B*, respectively. Note that this experiment cannot determine the 3D direction of the π-orbital orientation because the experiment was aimed at detecting the projection component of the orientation vector on the sample surface. In this study, a sample portion at which the π-orbital was relatively facing in-plane was sampled out and fabricated as a thin film. The direction of separation or cracking observed in each sample area was perpendicular to the π-orbital direction. This is said to be reasonable if considering the bonding feature and because the direction of the π-orbitals is perpendicular to those of the graphene sheets. This shows that a chemical-structure mapping (especially in the direction of the π-orbital-oriented domain or graphene sheets) can be performed by combining cSTXM with the developed sample holder. This is not possible solely through microstructural observations using TEM.

## 4. Conclusion   

In the study, an azimuthal-rotation sample holder was developed for an STXM. The holder exhibited improvements in the accuracy of the rotation angle and deflection of the surface of the disk plate. To evaluate the performance of the holder, the π-orbital orientation domains in carbon materials were investigated. The average orientation angle in each π-orbital oriented domain in natural spheroidal graphite was determined based on the dependence of the π^*^/σ^*^ ratio on the polarization angles of the X-rays using sample rotation and two orthogonal polarizations.
